# Experiences of mobile health in promoting physical activity: A qualitative systematic review and meta-ethnography

**DOI:** 10.1371/journal.pone.0208759

**Published:** 2018-12-17

**Authors:** Daniel D. Carter, Katie Robinson, John Forbes, Sara Hayes

**Affiliations:** 1 School of Allied Health, University of Limerick, Castletroy, Limerick, Ireland; 2 Health Research Institute, University of Limerick, Castletroy, Limerick, Ireland; 3 Graduate Entry Medical School, University of Limerick, Castletroy, Limerick, Ireland; Leibniz Institute for Prevention Research and Epidemiology BIPS, GERMANY

## Abstract

**Objective:**

Despite evidence supporting physical activity in primary and secondary prevention, many individuals do not meet recommended levels. Mobile health is a field with a growing evidence base and is proposed as a convenient method for delivering health interventions. Despite qualitative exploration of stakeholder perspectives, there is a lack of synthesis to inform evidence-based design. This study aims to resolve this by identifying and synthesising qualitative research on the experience of using mobile health applications to promote physical activity.

**Method:**

A systematic review focused on qualitative research, mobile health and physical activity was conducted in October 2017 using CINAHL, ERIC, EMBASE, MEDLINE and PsycINFO databases. The protocol was registered with the Prospero database (Registration: CRD42018080610). Results were synthesised as a meta-ethnography.

**Results:**

Fifteen studies were included, covering a variety of populations, including people with diabetes, obesity, and serious mental illness. Five themes emerged: (a) personal factors and the experience of using mobile health, (b) mobile health and changes in thinking that support physical activity, (c) the experience of mobile health features, including prompts, goal setting and gamification, (d) the experience of personalised mobile health and physical activity, (e) technical and user issues in mobile health and their effect on experience.

**Conclusion:**

Personal factors and features of the device influenced the experience of using mobile health to support physical activity. The two mechanisms through which mobile health use facilitated physical activity were strengthening of motivation and changes in self-awareness and strategising. Experiences were not entirely unproblematic as technical issues and adverse effects related to self-monitoring were noted. This synthesis provides insight into the experience of mobile health and is useful for researchers and healthcare practitioners interested in designing user-informed mobile health interventions for promoting physical activity.

## Introduction

The uptake of physical activity (PA) and exercise is a cornerstone strategy of primary and secondary prevention for non-communicable conditions and has long been a challenge for healthcare providers across disciplines and settings [1, 2]. Moderate to vigorous PA is associated with reduced risk of metabolic syndrome [3], cardiovascular disease [4] and all-cause mortality [5]. In addition to contributing to non-communicable disease, the economic burden of physical inactivity is significant, adding to healthcare costs and productivity loss [6]. Predictably, PA promotion is targeted at global [7], national [8–10] and illness-specific levels [11, 12]. Despite PA being a modifiable risk factor, PA guidelines are frequently unmet in the general population [13] and in illness-specific conditions post event, for example, in people with stroke [14].

Taking stroke as an illustrative example, a large international case-control study established association between PA and reduced risk of first stroke [15]. Given the established risk of recurrent stroke [16], clinical guidelines advocate the promotion of PA [11, 12]. These guidelines are supported by a strong evidence base, with a recent Cochrane review demonstrating cardiorespiratory training improved scores on global indices of disability (standardised mean difference (SMD) 0.52, 95% confidence interval (CI) 0.19 to 0.84; P value = 0.002) [17]. Step count is one method for approximating PA. For stroke survivors, daily step count is estimated at 4355.2 [18]. This falls below guidelines for the general population (10000) [19] and for adults with chronic illness (6500–8500 steps) [20]. Given the prevalence of physical inactivity in both illness-specific and the general populations in the face of evidence-based guidelines, novel approaches for promoting PA are required.

Tailored interventions are increasingly seen as a means for delivering care to individuals with chronic conditions [21, 22]. Tailoring refers to health material consisting of “any combination of information and strategies intended to reach one specific person that are based on characteristics unique to that person, related to the outcome of interest, and derived from an individual assessment” [21]. Tailored interventions have been successfully applied directly to PA through print-based [23] and internet-based interventions [24] in adult populations. It follows that mHealth may provide the next iteration of tailored interventions for PA. Though initial results have been mixed [25, 26], mHealth arguably is ideally placed to offer tailoring. Advances in mobile and sensing technology are now able to deliver just-in-time adaptive interventions which can offer the right type of support, at the right time, by adapting to individuals’ changing states [27]. More recently, wearable activity monitors paired with machine learning algorithms are being used to explore novel approaches to personalising PA interventions [28]. The use of wearable activity monitors is also notable as they offer objective reports of PA. This is vital to planning and measuring the efficacy of interventions, particularly as individuals are typically poor estimators of their own PA [29, 30]. Thus, interventions with automated monitoring can offer more reliable and valid reports of PA and are well poised for use in health interventions.

Mobile Health (mHealth) is a growing field defined by the use of portable devices including phones and tablets to improve health status [31]. The mHealth movement has been spurred by increasing smartphone ownership. A recent survey in the United States noted almost three quarters of adults report smartphone ownership, with similar levels reported in high and middle-income countries and growth on the rise in low-income countries [32]. Smartphones provide platforms for delivery of interventions which can bridge gaps between services or act as adjunct treatments [31]. A subsegment of mHealth has focused on PA promotion using applications. With thousands of ‘Health and Fitness’ applications in the iTunes and Google Play stores, there is significant variation in content and underlying theory, with many employing only minimal behaviour change techniques [33, 34]. In view of this, calls for an increase in theory-based applications have been made [35].

Similarly, the risk of overengineered solutions in mHealth has recently been highlighted alongside calls for the inclusion of end-user perspectives [36, 37]. To this end, the World Health Organization has included stakeholder involvement in their recently created mHealth evidence reporting and assessment checklist [31]. End-users’ experiential knowledge provides unique insight into the success or failure of interventions and supports transparency and legitimacy [38]. Several factors have been identified as influencing engagement with digital health interventions, including personal agency, motivation and prior experience of using mobile devices [39]. Additionally, features common to mHealth, like self-tracking, have been described as polarising, with some suggesting they afford opportunities for empowerment and others critiquing their ability to induce feelings of anxiety or to infringe on users’ privacy [40–42]. By better understanding end-users’ perspectives on mHealth in the context of PA promotion, improvements can be made in the development and implementation of future interventions.

Qualitative research is well placed to elicit the user’s perspective and can contribute constructively to intervention development, with meta-ethnography specifically noted as a useful method for synthesising qualitative literature in health technology assessment [38]. Meta-ethnographic approaches have previously yielded valuable findings in exploring user experience of computerised therapy for depression and anxiety [43], telehealth user experience for chronic obstructive pulmonary disease [44] and experiences of self-management support following stroke [45].

The current systematic review and synthesis will form a part of a larger project to design a mHealth application-based intervention to promote PA in adults with stroke. Medical Research Council guidance on designing complex health interventions recommends identifying the existing evidence base as part of the initial stages of development [46]. While reviews have previously been completed on this topic, they have focused on the quantitative literature [25, 26] or a mix of quantitative and qualitative literature [47, 48], with the efficacy of applications aimed at promoting PA noted to be mixed.

The aims of the study were to:

1. Systematically search the qualitative literature to identify studies exploring the experience of adults using mHealth to promote PA.
2. Perform a meta-ethnography to synthesise the included studies with a view to identifying new insights and describing user experience.

## Method

### Design

A meta-ethnographic synthesis, informed by Noblit and Hare’s [49] seven-stage process, of the qualitative literature was selected to move beyond collation of the evidence base and toward generation of new understanding [50]. The original protocol can be accessed using the Prospero database (Registration: CRD42018080610). It is reported in accordance with the Enhancing Transparency in Reporting the Synthesis of Qualitative Research (ENTREQ) guidelines [51] (S1 File).

### Search strategy

A systematic search of CINAHL, EMBASE, ERIC, MEDLINE and PsycINFO was completed using “qualitative research”, “PA” and “mHealth” as keywords alongside thesaurus and MeSH terms in October 2017. These databases were selected for their inclusion of qualitative studies and health research. The keywords used in the search strategy were drawn from recently conducted systematic reviews for qualitative research [52], PA [17] and mHealth [53] (S2 File). Those key words were validated and additional key words added by checking the terms used in articles identified in preliminary searches. No limit was placed on date of publication. Studies were limited to those published in English and those involving adults.

### Inclusion and exclusion criteria

Studies were included if they reported qualitative research which focused on the experience of adults who had used mHealth applications alone or mHealth applications and wearable activity monitors to promote PA in day to day life, as opposed to a laboratory or experimental setting (e.g. trialling the usability of a prototype mHealth application). Studies were also included if they reported using mixed methods or if they reported on the views of healthcare providers or other stakeholders provided qualitative data regarding the end-users could be extracted separately.

### Screening

Titles and abstracts were screened independently against inclusion criteria by two reviewers (DC, KR or SH). Each record was screened independently twice, with disagreements resolved through discussion and consensus with a third reviewer where necessary. Full text articles were screened by two reviewers (DC, KR) for final decisions regarding inclusion, with disagreement resolved by consulting a third reviewer (SH).

### Quality appraisal

The methodological quality of the included studies was appraised using the ten-item Critical Appraisal Skills Programme (CASP) checklist for qualitative research [54]. The CASP tool, is widely used in qualitative research despite its limitations [55] and has been recommended for use in health research [56]. Two evaluators (DC, KR) independently assessed the quality of each study with discrepancies resolved through consensus and discussion with a third evaluator (SH).

### Data extraction and synthesis

The synthesis was informed by the seven phases of meta-ethnography originally described by Noblit and Hare [49]. This is one of the most commonly used methods of qualitative synthesis [57] and is interpretative rather than integrative or aggregative, focusing on the generation of new understanding [58]. The focus of the analysis was synthesising themes or third-order constructs from second-order constructs (themes identified by the authors of included studies) [49].

The first phase, ‘getting started’, involved development of a research question and the second phase, ‘deciding what is relevant’ involved searching for and quality appraising each included article. In phase three, ‘reading the studies’, articles were closely read by two researchers who extracted second-order concepts into QSR International’s NVivo 11 Software. In phase 4, ‘determining how the studies are related’, a grid of concepts was developed using the extracted data. Concepts from each study were juxtaposed against one another to lay the foundation for phase five, ‘translating the studies into one another’. In phase six, ‘synthesising translations’, a line-of-argument was synthesised from third-order constructs. The line-of-argument represents what can be said “of the whole… based on selective studies of the parts” [49]. In the current study, the whole refers to the experience of using mHealth to promote PA. The final phase ‘expressing the synthesis’ was achieved through writing up the results for dissemination. Phases four through six were led by DC, with critical feedback provided throughout by KR.

## Results

### Search outcomes

**Fig 1:**
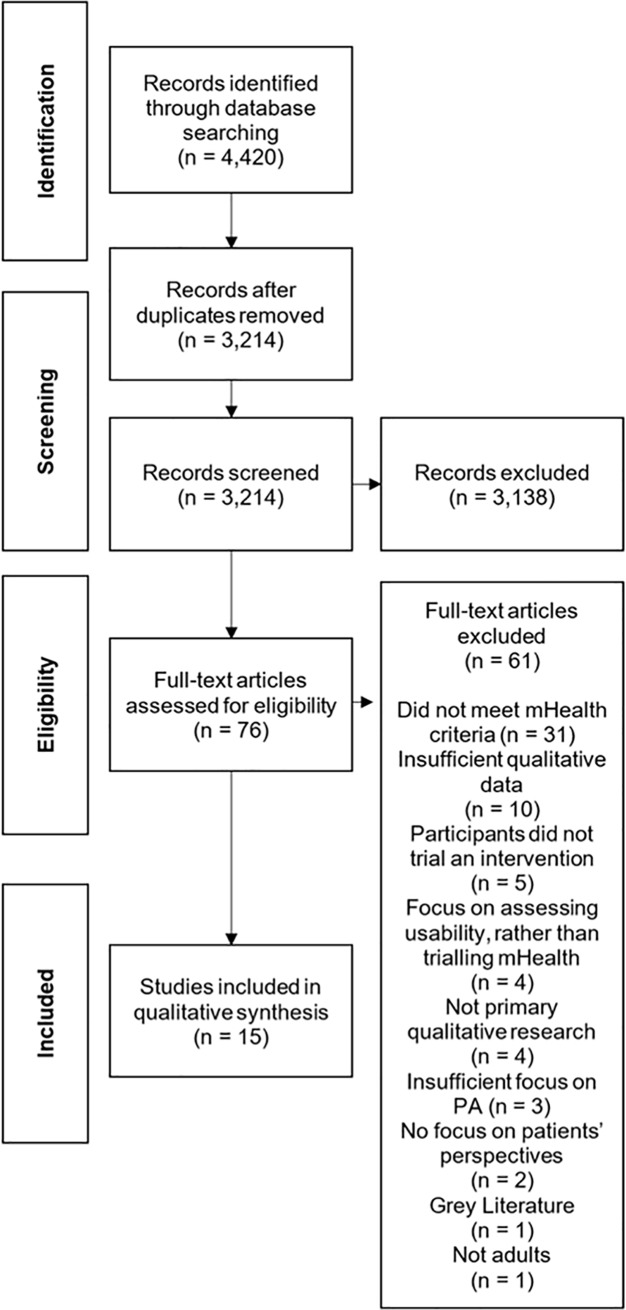
PRISMA flow diagram.

In total, 4420 studies were identified and removal of duplicate studies left 3214 studies for screening. Titles and abstracts were read and a further 3138 articles were removed based on the selection criteria. Seventy-six full text articles were screened. Fifteen studies met the criteria for inclusion and were included in the meta-ethnography (Fig 1).

### Characteristics of included studies

The studies included were diverse and details of each are included in Table 1. Six studies were conducted in the United States [59–64], two in Australia [65, 66] and the Netherlands [67, 68] and one each in Canada [69], Norway [70] and the United Kingdom [71]. Two comparative studies took place in the United States and Sweden [72] and in Finland and India [73]. With regards population, nine studies were conducted with patient groups [59–61, 63, 64, 66, 68–70] and six studies recruited community dwelling participants [62, 65, 67, 71–73]. Participants ranged in age from 18 [62, 65–67, 71] to 75 [69]. Three studies included only young adults [62, 66, 67], two studies included young and middle aged adults [71, 73], six included middle aged and older adults [59, 60, 63, 68–70] and three included young, middle aged and older adults [61, 64, 65], while one study did not report age [72]. Two studies [59, 67] reported the behaviour change techniques used in their applications and two studies [63, 70] reported objective PA data.

**Table 1:** Characteristics of included studies.

Citation and setting	Population	Sample n (n females), age in years	Research aim(s)	Methodology (refers to overall design in studies with multiple phases, data collection and data analysis	mHealth component, application content, behaviour change techniques and duration	Additional support	Physical activity data	Summary of findings (not all studies generated themes)
Ahtinen et al 2013 [73] Finland, India	Adults living in urban areas, engaged in sedentary work and interested in wellness management Prior mHealth experience: All participants were active mobile phone users.	Two studies reported on. Only Study 1 included. Study 1 total: 16 (9) Finnish participants: 8 (4) Mean: 33 Range: 25–45 Indian participants: 8 (5) Mean: 36 Range: 25–50 Young and middle-aged adults	To study users’ needs in relation to a mobile wellness application that supports engagement in PA and to analyse those findings and formulate user-centric design principles for a mobile wellness application to motivate people to exercise.	Qualitative Interview Content analysis	Off the shelf application (Wellness Diary), some participants received a smartphone (Nokia 5500 Sport) if their own phone was incompatible with the application. Wellness Diary allows users to maintain a journal of wellness parameters for their own goals, including weight, exercise, eating, and others. The application offered feedback through lists and graphs. BCTs not reported. Duration: 2 weeks	mHealth application installed on participants’ phones and an introduction to its use was provided.	PA not reported objectively or self-reported. Baseline PA level not noted as inclusion criteria, but participants were described as not “completely inactive or extremely active”. Participant motivation for taking part in study not reported.	Four main themes identified: (1) getting bored, (2) beyond numbers, (3) interaction and variety, and (4) advisory role.
Anderson, Burford and Emmerton 2016 [65] Australia	Consumers of mHealth applications, some with chronic health conditions Prior mHealth experience: All participants had used mHealth prior to interviews (Only participants with application use were recruited).	22 (15) 17 participants reported using fitness applications, one reported using a cycling application. Remaining participants used a variety of self-care applications. Range: 18–55+ (Range for users of PA applications not specified) Young, middle aged and older adults	To explore health consumers’ use of applications for health monitoring, perceived benefits from use of health applications, and suggestions for improvement of health applications.	Qualitative Semi-structured interview Deductive and inductive thematic analysis	Multiple off the shelf applications 22 different fitness applications and 2 different cycling applications were used. BCTs not reported. Duration: 2 weeks to 2+ years	None	PA not reported objectively or self-reported. Baseline PA: Not applicable, participants were already using applications at time of interview (naturalistic use). Motivations for application use included: greater self-awareness of one’s condition, easier self-management, ability to share data with healthcare professional, ability to view historical health data, social motivation to improve fitness and greater control over their condition.	Four main themes were generated: (1) engagement in use of the application, (2) technical functionality of the application, (3) ease of use and design features, and (4) management of consumers’ data.
Årsand et al 2010 [70] Norway	Type II Diabetes Prior mHealth experience: Not reported.	12 (8) Mean: 56.2 Standard deviation: 9.6 Range: 44–70 Middle aged and older adults	To explore how patient-operated self-management tools can be designed for supporting lifestyle changes among people with type 2 diabetes and how these tools were perceived by a group of 12 patients during a 6-month period.	Mixed methods Focus group Not specified	Custom application (Few Touch Application), OneTouch Ultra 2 blood glucose monitor and PA sensor system using Bluetooth. The Few Touch Application supports adults in managing their diabetes. It records blood glucose, step count and food habits and provides feedback in relation to users’ goals. BCTs not reported. Duration: 6 months. PA component introduced late and tested for 2–3 months.	No additional support reported.	PA reported only objectively (average step count in first and final weeks). Baseline PA not reported or required as inclusion criteria. Participant motivation for taking part in study not reported.	User feedback from the intervention demonstrated good usability of the tested system, and several of the participants adjusted their medication, food habits, and/or PA.
Bentley et al 2013 [72] United States, Sweden	Adults recruited via a professional recruitment agency and through the researchers’ extended social networks Prior mHealth experience: Not reported.	Pilot study 10 (not reported) Full study 60 (not reported) Age not reported.	To develop, trial and evaluate a system that supports reflection on personal wellbeing data and context.	Mixed methods Semi-structured interview, voicemail and email for pilot. Semi-structured interview and questionnaires for full study Grounded theory	Custom application (Mashups application), wearable activity monitor (Fitbit) and Internet-connected scale (Withings) Health Mashups aims to increase self-understanding and behaviour change by identifying significant connections between weight, sleep, step count, calendar data, location, weather, pain, food intake and mood. BCTs not reported. Duration: 2-month pilot study; 90-day full study	In both studies, researchers set up the scale, Fitbit and Mashups application and demonstrated their use. Participants were questioned about their wellbeing and goals at the start of each study. Participants were told that using the Mashup application was not required to take part in either study.	PA not reported objectively. Self-reported increase in PA noted. Baseline PA not reported or required as inclusion criteria. Participant motivation for taking part in study not reported.	Findings from the pilot study were presented as four categories: (1) learning from observations, (2) lack of data richness, (3) contradictory information, and (4) design recommendations. Findings from the full study were presented as four categories: (1) sustained use, (2) increased self-understanding, (3) behaviour change, and (4) obvious observations.
Buman et al 2016 [59] United States	Veterans with increased metabolic risk Prior mHealth experience: Participants required to have a smartphone.	Total study: 26 (4) Mean: 49, Standard deviation: 8.9 Range: 36–65 Focus group: 17 (Due to randomisation of participants, it was unclear how many were exposed to each components) Middle aged and older adults	To develop and complete a process evaluation of BeWell24, a multicomponent application targeting lifestyle behaviour: sleep, sedentary and active behaviour.	Mixed methods Structured interview with open-ended questions Case study approach	Custom application (BeWell24) BeWell24 targets behaviour change in sleep, sedentary behaviour, and physical activity. BCTs reported in the sedentary behaviour and exercise component of application: Feedback and monitoring, natural consequence, associations, repetition and substitution, goals and planning, and shaping knowledge. Duration: 8 weeks (3-week run-in period where only the self-monitoring component was available)	Participants attended two additional visits during the 8 weeks to complete study-related assessments.	PA not reported objectively. Self-reported increase in PA noted. Baseline PA not reported. Inclusion criteria included self-reported insufficient PA (endorsing activity ranking categories ≤4 on the Stanford Brief Activity Survey) and excessive sitting (≥8 h of sitting from the International Physical Activity Questionnaire). Participant motivation for taking part in study not reported.	Findings were related to each of the application’s components, with themes noted in each. Three themes related to self-monitoring: (1) awareness, (2) ease of use, and (3) time spent using the self-monitoring component. Three themes related to the behavioural application component (i.e., sleep, sedentary, and PA): (1) content, (2) awareness/motivation or behaviour, and (3) modifications/ recommendations. No clear themes emerged from the exercise component.
Eisenhauer et al 2017 [60] United States	Rural men (Overweight/ obesity and prehypertension/ hypertension noted in sample) Prior mHealth experience: Participants had to possess a personal computer and be able to send and receive text messages. Those who had used a Fitbit or similar (excluding pedometer) previously were excluded.	12 (0) Mean: 50.9 Standard Deviation: 8.6 Range: 40–67 Middle aged and older adults	To examine the feasibility and acceptability of health-related text messages and use of a wearable activity monitor and companion application to self-monitor eating and activity as perceived by rural men.	Mixed methods Focus group Descriptive content analysis	Off the shelf mHealth application (Fitbit One companion application), wearable activity monitor (Fitbit One) and text messages. Fitbit One wearable activity monitor used to track PA and Fitbit One companion application used to record diet and monitor PA. Participants could monitor the PA of peers in the study through the application. BCTs not reported. Duration: 3 weeks	Brief orientation provided to explain mHealth components.	PA not reported objectively. Self-reported increase in PA noted in a survey administered after the intervention. Baseline PA not reported or required as inclusion criteria. Participant motivation for taking part in study not reported.	Self-monitoring and daily text messages increased awareness of energy intake and output. Fitbit One and text messages were perceived as useful, while the companion application requires adaptation to reflect dietary norms.
Fukuoka, Lindgren and Jong 2012 [61] United States	Sedentary women Prior mHealth experience: Not required or reported.	41 (41) Mean 48.4 Standard deviation: 13.1 Range: 25–70 Young, middle aged and older adults	To explore acceptability and understand motivators and barriers to increasing PA using a mobile phone application and pedometer.	Qualitative Semi-structured interview Thematic analysis	Custom application, mobile phone (MOTORAZRv3xx) and pedometer (Omron HJ-720 ITC) The custom application delivered daily messages, set automated weekly goals, provided immediate feedback and self-monitoring functions (mobile diary), and collected participant responses. BCTs not reported. Duration: 3 weeks	Brief face-to-face intervention consisting of: (1) an overview of the program, (2) education regarding PA, (3) counselling regarding barriers to PA, (4) increasing social support, and (5) safety for PA.	PA not reported objectively. Self-reported increase in PA noted. Baseline PA: Sedentary lifestyle at work and/or during leisure time (Brief Physical Activity Survey questionnaire) required Participant motivation for taking part in study not reported.	Findings identified three main themes: (1) monitor me: mobile phone/ pedometer as self-monitoring tools, (2) motivate me: cycle of feedback in goal setting and usefulness/uselessness of daily random messages, and (3) mobilise me: engaging and adapting PA to fit one’s own lifestyle.
Gowin et al 2015 [62] United States	University students Prior mHealth experience: All participants had mHealth experience (no intervention, only participants with application use were recruited).	27 (21) Mean: 20 Range: 18–30 Young adults	To describe how college students in the Southwestern United States use health and fitness applications to change behaviour.	Qualitative Semi-structured interview Grounded theory	Multiple off the shelf applications Various health/ fitness applications used BCTs not reported. Duration: 1 month to 1+ year	None	PA not reported objectively Self-reported increase in PA noted. Self-reported times spent exercising per week reported at time of focus group. Baseline PA: Not applicable, participants were already using applications at time of interview (naturalistic use). Motivations for application use were classified after interviews as either adopting a new behaviour or maintaining/ improving an existing behaviour.	Findings were presented as three main themes: (1) acquiring the application, (2) utilizing the application, and (3) likes/ dislikes about the application.
Knight and Petrella 2014 [69] Canada	Primary care patients Prior mHealth experience: Not reported. Some participants self-reported not having mHealth experience in results.	20 (12) Mean 63 Standard deviation: 5 Range: 55–75 Middle aged and older adults	The aim was to perform a longitudinal follow up on a PA and mHealth intervention at six months. The study aimed to: (1) Determine if improvements made through the intervention phase were maintained long-term by measuring a clinical marker of cardiometabolic health risk at 6 months postintervention, and (2) to elicit themes describing participants’ experiences in a program aimed to modify lifestyle using activity prescription and mHealth.	Mixed methods Semi-structured interview Whole text analysis	Not specified whether application is off the shelf or custom. Application provided with technology kit (smartphone, blood pressure monitor, glucometer, pedometer and weight scale). The application was used to manage submitted measures (PA, blood pressure, blood glucose, and body weight), providing tabular and graphical feedback. BCTs not reported. Duration: 12-week intervention, participants followed up 6 months post intervention	Preintervention visit: activity prescription included counselling with a certified exercise physiologist and a personalised activity program. Postintervention visit: Written prescription indicating their VO2max, target training heart rate, and amount of activity required to meet PA guidelines provided.	PA not reported objectively. Baseline and final average functional aerobic capacity reported. Some participants self-reported sustaining their PA levels prescribed during the intervention. Baseline PA not reported or required as inclusion criteria. Participant motivation for taking part in study not reported.	Three emergent themes were noted: (1) desire for short-term mHealth intervention to educate individuals about prescribed health behaviours without need for ongoing management by clinicians, (2) leveraging mHealth to build social networks around prescribed health behaviours and to connect individuals to build a sense of community, and (3) participant views of PA as medicine.
Lewis et al 2017 [63] United States	Primary care patients Prior mHealth experience: Not reported.	Total study: 40 (30) Mean: 63.7 Standard deviation: 5.3 Range: 55–74 Electronic activity monitor group with application: 20 (17) Mean: 64 Standard deviation: 5.1 11 participants took part in focus group and only 8 had used an activity monitor and companion application. No demographic data was provided at this level. Middle aged and older adults	To determine the feasibility and acceptability, using the RE-AIM (Reach, Effectiveness, Adoption, Implementation, Maintenance) framework, of a primary care-based intervention that incorporated 5 A’s counselling (assess, advise, agree, assist, and arrange) and self-control through an activity monitor.	Mixed methods Focus group, interview Thematic analysis	Off the shelf application (Jawbone UP companion application) with wearable activity monitor (Jawbone UP24) or pedometer. The Jawbone UP24 wearable activity monitor was used to track PA and the companion application allowed participants to monitor their PA, diet and weight, as well as interact with other participants. BCTs not reported in relation to application. The authors noted that wearable activity monitors could support BCTs, including: action planning, cues to action, social support, and learning from peers. Duration: 12 weeks	Exercise counselling and prescription prior to randomisation to pedometer or activity monitor groups.	PA recorded objectively (time spent doing moderate or vigorous exercise increased). No self-reported PA data. Baseline PA: Physically inactive (self-reported less than 60 min/week of planned PA), Participant motivation for taking part in study not reported.	Findings centred on four main themes: (1) testing activity monitors’ effect on health, (2) self-monitoring, (3) social support on the UP app, (4) counselling from the counsellor or from a health care provider. Over the 12 weeks, there were 490 comments and 1094 “likes” given to study peers in the companion application. Some activity monitor participants enjoyed the social interaction while others were uncomfortable talking to strangers.
Middelweerd et al 2015 [67] Netherlands	University students Prior mHealth experience: Participants had to own a smartphone with Internet access.	30 (20) Mean: 21 Standard deviation: 2 Range: 18–25 Young adults	To explore Dutch students’ preferences regarding a PA mHealth application.	Qualitative Focus group Conventional content analysis	Off the shelf application (Nexercise) Nexercise is a fitness tracker that supports GPS tracking, an activity log book, earning points, a competition feature, chat features and linking with social media. BCTs reported: Prompting goal setting, prompting self-monitoring, providing feedback on performance, providing rewards and planning social support. Duration: 3 weeks	Participants were asked to use the application and to share accomplishments on social media. Neither using the application, nor sharing on social media were required to take part in the focus groups.	PA not reported objectively. Baseline PA not required as inclusion criteria. Baseline PA: Participants were divided into those did and did not meet Dutch PA guidelines (Dutch short version of the International Physical Activity Questionnaire). Participant motivation for taking part in study not reported.	Findings were presented in five main categories: (1) general application usage, (2) technical aspects, (3) PA assessment, (4) coaching aspects, and (5) sharing through social media.
Morrison et al 2014 [71] United Kingdom	Adults recruited from a university campus with no pre-existing health condition which might impede nutrition or PA modification. Prior mHealth experience: Participants required to own an Android mobile phone.	13 (7) Median: 27 Range 18–52 Young and middle aged adults	To examine individual variation in (1) impact on self-reported goal engagement of access to a weight management application when provided alongside a Web-based weight management intervention and (2) usage and views of a mHealth application.	Mixed methods Semi-structured interview Inductive thematic analysis	Custom application (POWeR Tracker) POWeR Tracker provides informational tools (viewing goals, plans and content from the intervention web site) and self-monitoring tools (personalised feedback on goal progress). BCTs not reported. Duration: 7 weeks total (3 weeks with website and 4 weeks with website and mHealth application)	Participants had access to the Positive Online Weight Reduction (POWeR) web-based weight management intervention which was delivered over 12 sessions. Access to the POWeR Tracker application was alternated on a weekly basis.	PA not reported objectively or self-reported. Baseline PA not reported or required as inclusion criteria. Participant motivation: The authors noted all participants were motivated to adopt a healthier lifestyle.	Four main themes were generated from the interviews: (1) convenience and accessibility, (2) constant reminder and repetition, (3) motivational benefits of tracking, and (4) time-relevant use guided by lifestyle and routine.
Naslund, Aschbrenner and Bartels 2016 [64] United States	Serious mental illness and obesity Prior mHealth experience not reported. Some participants self-reported not having mHealth experience in results.	11 (8) Mean: 48.2 Standard deviation: 11.2 Range: 21–57 Young, middle aged and older adults	To assess acceptability of smartphones and wearable devices to support a lifestyle intervention targeting weight loss in adults with serious mental illness.	Mixed methods Semi-structured interview Rapid content analytic approach	Off the shelf application (Fitbit companion application) and wearable activity monitor (Fitbit Zip) The Fitbit Zip recorded steps, distance, active minutes and calories burned. The companion application tracks progress, rewards milestones with colourful trophies, and lets participants set daily step goals, as well as compare their progress with peers. BCTs not reported. Duration: 6 months	Participants were enrolled in a 6-month lifestyle intervention adapted from the Diabetes Prevention Program curriculum and delivered through a community mental health centre.	PA not reported objectively. Some participants self-reported increases in PA. Baseline PA not reported or required as inclusion criteria. Participant motivation for taking part not reported.	Three main themes were identified: (1) motivating, encouraging, fun to use and other benefits, (2) other things the Fitbit can do, and (3) technical difficulties, challenges and recommendations for improvement.
Partridge et al 2016 [66] Australia	Young adults who failed to meet national exercise and nutrition recommendations Prior mHealth experience: Participants must have a mobile phone capable of receiving text messages and access to the Internet at least once weekly.	Total sample: 248 (152) Mean: 27.7 Standard deviation: 4.9 Range: 18–35 Interviewed: 30 (17) Interview: Age not specified Young adults	To investigate participants’ perceptions of and engagement with the mHealth program components in the TXT2BFiT to understand program effects.	Mixed methods Semi-structured interview Content inductive analysis	Custom applications and text messages. Four separate applications were developed, however, their functions beyond self-monitoring were not reported. BCTs not reported (authors noted that collectively their seven applications contained 18 BCTs). Duration: 9 months, [3-month intervention and 6-month maintenance phase]	The TXT2BFiT program was a multi-component lifestyle intervention delivered intensively for 3 months and followed by a 6-month maintenance phase. Program components included personalised coaching calls, text messages, emails, smartphone applications and website access.	PA not reported objectively or self-reported Self-reported PA below national guidelines noted as inclusion criteria. Participant motivation for participation included: weight loss and being more physically active.	Results related to website and application use noted engagement was low for the duration of the program. Participants would have preferred incorporation of the self-monitoring applications and website resources into one smartphone application that could be individualised by entry of their personal data.
van der Weegen et al 2014 [68] Netherlands	People with chronic obstructive pulmonary disease or type 2 Diabetes Prior mHealth experience: Not reported	Four phases, but only phase 3 involved use in real life and is reported on here. Phase 3: 20 (9) Mean: 60.2 Standard deviation: 9.0 Middle aged and older adults	The aim was to improve the user interfaces and content of It’s LiFe!, a monitoring and feedback tool to stimulate PA.	Mixed methods Interview Directed content analysis	Custom application (It’s LiFe! application), smartphone (Samsung Galaxy Ace) and activity sensor. It’s LiFe! application provides real-time feedback on PA in relation to personal goals and provides dialogue sessions about PA barriers and facilitators. BCTs not reported. Duration: 3 months	Three practice nurse consultations (before, during and after the trial). Participants also received dialogue sessions via the application and could access a website with questions about barriers and facilitators to PA.	PA not reported objectively or subjective (baseline data was recorded for two weeks for goal setting). Baseline PA not reported or required as inclusion criteria. Participant motivation for taking part in study not reported.	The findings from phase 3 were presented as five categories: (1) application usage, (2) technical aspects, (3) PA assessment, (4) coaching aspects, and (5) sharing through social media.

BCT, behaviour change technique; mHealth, mobile health; PA, physical activity; young adult = 18–35; middle aged = 36–55; older adult = 56+

### Quality appraisal

The quality of studies included varied. As noted in Table 1 above, several studies employed exclusively qualitative methods, while others employed mixed methods. In some instances, studies reported their findings from multiple phases [68, 72] or a multicomponent intervention where mHealth application was part of a wider, multicomponent study [60, 61, 63, 68, 70, 71].These factors may have compromised reporting of methods. Notably, only three of the studies attempted to address the relationship between researcher and participant [62, 64, 73]. Furthermore, two studies did not detail the rationale for or how they completed their analyses [69, 70]. However, given the lack of agreement on the application of quality criteria [74], no studies were excluded on the basis of quality. A summary of the results from the CASP tool are provided in S1 Table.

### Synthesis

The analysis produced five themes (third-order constructs): (a) Personal factors and the experience of using mHealth, (b) mHealth and changes in thinking that support PA, (c) the experience of mHealth features, including prompts, goal setting and gamification, (d) The experience of personalised mHealth and PA, (e) technical and user issues in mHealth and their effect on experience. An overview of each study’s contribution to the themes is provided in Table 2.

**Table 2:** Contribution of included studies towards themes.

Citation	Personal factors and the experience of using mobile health	Mobile health and changes in thinking that support physical activity	The experience of mobile health features, including prompts, goal setting and gamification	The experience of personalised mobile health and physical activity	Technical and user issues in mobile health and their effect on experience
Ahtinen et al 2013 [73]	X		X	X	X
Anderson, Burford and Emmerton 2016 [65]	X	X	X	X	X
Årsand et al 2010 [70]		X	X		X
Bentley et al 2013 [72]	X	X	X	X	X
Buman et al 2016 [59]	X	X	X	X	X
Eisenhauer et al 2017 [60]		X	X		X
Fukuoka, Lindgren and Jong 2012 [61]		X	X	X	
Gowin et al 2015 [62]	X	X	X		X
Knight and Petrella 2014 [69]	X	X	X	X	X
Lewis et al 2017 [63]		X	X		X
Middelweerd et al 2015 [67]	X	X	X	X	X
Morrison et al 2014 [71]		X	X	X	X
Naslund, Aschbrenner and Bartels 2016 [64]	X	X	X	X	X
Partridge et al 2016 [66]	X	X			X
van der Weegen et al 2014 [68]			X		X

### Personal factors and the experience of using mHealth

Personal factors were reported in multiple studies to shape engagement with and experience of using mHealth, particularly users’ prior experience with and rationales for using mHealth. Personal factors influenced participants’ motivation to engage with applications.

Prior experience was noted to be influential in two ways. Limited prior experience of mHealth components, including smartphones, applications and wearables, was noted in a segment of participants and slowed initial engagement. This was noted in three studies [59, 64, 69], but generally appeared not to be insurmountable:

Understanding how it worked. It was a little difficult. I’m not too fast on technology; it takes me a long time to use technology. When it comes to these phones, they’re a little bit more sophisticated than some of the stuff that I use. It was a challenge. [64]

Conversely, participants in Partridge and colleagues’ [66] study had prior experience with mHealth applications, which appeared to result in high expectations. Some of their participants abandoned the researchers’ applications and reverted to commercial applications which better suited their needs:

I decided that I already have apps on my phone that I use to track diet and exercise, so I kept using those ones…. I think it was the usability of the TXT2BFiT ones, especially trying to use [the apps] on my phone, I found them a bit difficult to navigate. [66]

Personal motivations or reasons for application use also influenced engagement. Two studies explored differences between those initiating and those maintaining PA behaviour [62, 67]. Gowin and colleagues [62] divided participants by national PA guidelines. Those who did not meet the PA guidelines expressed preference for an application taking on a coaching role which could provide encouragement and a training schedule with tasks to complete. Those who met PA guidelines preferred a coaching feature which guided them to intensify existing training sessions. These findings were supported by Middelweerd and colleagues’ [67] analysis. Though their study did not separate participants by level of PA, they noted in their data that those with established healthy behaviours reported looking for ways to support that behaviour, to make it easier or to target specific factors related to it. Those trying to adopt a new behaviour had failed at it at least once before downloading the application. These participants looked for help with changing their behaviour, e.g. looking for support with adopting a new exercise routine. Similarly, Anderson, Burford and Emmerton [65] reported how some of their participants were interested in using an application to achieve a specific goal and ceased or decreased use once their goal was achieved. Other rationales for longer-term adherence were to monitor personal data for themselves [65, 67, 72] and to share personal data with healthcare professionals [65, 69]. Gowin and colleagues [62] reported participants who discovered an application from a family member usually used the application alongside them and reported positive feelings about same.

It would be remiss to not note that participants in three studies expressed disinterest in using applications for the promotion of PA [59, 67, 69]. One study noted a disinterest in mHealth applications and a preference for print-based material [59], while another noted some participants felt applications might be helpful for others, but not themselves [67]. The third study was comprised of middle aged and older adults, with few participants reporting willingness to ongoing smartphone use to self-monitor their health [69].

Notably, Ahtinen and colleagues’ [73] comparative study highlighted differences based on their participants’ nationalities. Finnish participants reportedly placed more value on the quantified elements their application offered, like graphs and goal setting, than their Indian counterparts. One Indian participant summarised the experience by concluding that “[t]his application is symbolic of the Western attitude (towards wellness)” [73].

### mHealth and changes in thinking that support physical activity

The use of mHealth components was described by participants across the included studies as facilitating various changes in ways of thinking and self-awareness that supported PA. Although the focus of the included studies was not on measuring changes in PA, seven studies noted some participants self-reported increased PA after engaging with applications or wearables [59, 60–62, 64, 69, 72]. One additional study noted increases in objective PA data in some participants [70], while a second study noted participants in their electronic activity monitor group objectively increased their time spent engaged in moderate or vigorous PA [63].

One of the more ubiquitous experiences reported across studies was heightened awareness of PA because of application and wearable activity monitor use. Heightened awareness provided opportunities for reflection which aided attempts made by participants to adapt their routines or lifestyles to incorporate additional PA. However, self-monitoring was not perceived as positive in every instance.

Ten studies referenced heightened awareness of PA behaviours through a wearable activity monitor or application use [59–65, 67, 71, 72]. Responses to being made aware of their PA were mixed. Some participants had overestimated their PA level and were shocked by their inactivity: “[I] didn’t know that I was not as active as I thought I was. On the days when I didn’t run or walk I realized that I didn’t even cover a mile a day and was horrified” [72]. Other participants were positively surprised by how active their baseline was: “It surprised me how many miles a day I put on just at work” [60].

Three studies noted that their participants not only became more aware of their PA behaviour, but the applications helped facilitate opportunities for reflection [65, 71, 72]. Morrison and colleagues [71] noted reflection could prompt further goal-directed behaviour. Similar findings were noted by participants in Bentley and colleagues’ [72] study: “I am a grad student who is overwhelmed and [the observations] helped to reflect on my life. They allowed me to take inventory and think about what I should change” [72].

While self-monitoring was generally valued by participants, the potential for adverse effects was noted. In some instances, application use was associated with frequent checking behaviour [60], while other end-users reported an “obsession” or “unhealthy” preoccupation with the application [62]. Negative experiences triggered by not meeting goals ranged from being “discouraging” [59] to “guilt, avoidance, shame, or feeling stressed” [62]. One participant reported: “It’s definitely like… almost like a peer pressure feeling at first just because it’s got it right there in big bold letters that you kind of screwed up today” [62].

Problem solving, requiring conscious effort on the part of participants, appeared to be supported by applications. Five studies reported participants using applications to support strategising or problem solving around PA [59, 61, 62, 69, 72]. By examining trends in personal data over time, participants could target specific days in the week where their PA was lower [72].

The application used by Fukuoka, Lindgren and Jong [61] forwarded prompts via the application which prompted strategising:

I liked having the questions on how I was going to manage it. Even though sometimes they weren’t applicable to me, it made me start thinking. I enjoyed that because it made me think more about my walking during the day so that I increased steps. [61]

Thus, prompting strategising or problem solving via the application also promoted PA. Four studies noted participants’ use of applications or wearables supported the incorporation of PA into their routines and lifestyles [61, 62, 65, 72]. This process also appeared to require conscious effort and thinking by the end-user.

I really want to have a more active lifestyle… Being able to just look at [the smartwatch] on the fly and going, “Right, if it just means that I have to go move that little bit more, or I have to exercise that little bit more”, I will do it, because you have a real-time gauge of how well you've done for the day. So that gets me going because the perceived barrier of just getting the thing done is a lot lower. [65]

### The experience of mHealth features, including prompts, goal setting and gamification

Several studies credited application features with supporting participants’ motivation to engage in PA. Features included social features, prompts, goal setting, and gamification.

Four studies discussed social features which facilitated competition and support [60, 63, 65, 67], while mixed opinions regarding social media were noted in two [62, 67]. Some participants were motivated by comparison of their PA to their peers. Eisenhauer and colleagues [60] explored the attitudes of rural males towards mHealth. Their application monitored PA and facilitated comparisons between participants. Some participants valued the opportunity to compete and felt it could provide motivation to increase PA: “If you got a group of people that you knew and who knew everybody and it was a competition… Little brother would always want to outdo big brother” [60]. Other participants reported value in building a sense of community through mHealth devices with other participants in their study [69].

In other instances, competition which was met with mixed reactions. Some end-users found it unnecessary and, due to limited time, only wished to focus on their own exercise rather than on playing a game [67]. Still others noted it was ‘confrontational’ which led to both encouragement and discouragement of PA [67].

In addition to competition, some applications offered participants the opportunity for directly supporting one another. The application used by Lewis and colleagues [63] allowed users to “comment” and “like” each other’s activity. While not all participants availed of this, those that did found it beneficial:

If I saw that somebody had done a lot that day I would give them a thumbs up and stuff like that… and then other people would encourage me and I didn’t know who they were either but their icon. [63]

Social media was discussed in two studies. Gowin and colleagues [62] noted that although half of their participants reported they were not against it, none of their participants made use of the ability to share achievements from their mHealth applications via social media. Some reported disliking it when applications did this automatically: “Yeah you can share on Facebook and stuff, but I hate that. I hate when apps sync to like every form of social media. I’m like really weird about social media, so, no I don’t want to share it” [62]. Middelweerd and colleagues [67] reported some participants enjoyed feedback and “likes” on PA-related Facebook posts from peers, however, they also noted that participants would only share major achievements, e.g. running a marathon or winning a match, on social media. In their focus groups, the authors noted that participants would be more willing to share achievements within smaller, private groups which were formed around similar interests or goals.

Some applications offered ‘coaching’ features which were well received. Four studies noted that participants valued or would like a “coaching” or “personal trainer” feature to interact with [61, 62, 67, 73]. One wanted “[a] coach who really encourages you, who is saying that you are doing a good job and who tells you to see you the next time, that is really nice” [67]. Fukuoka, Lindgren and Jong [61] noted some participants desired something further than coaching, which the authors described as a “counselling” type of interaction. They desired that their application’s messages would support them:

I remember one time I had like the highest I ever had and I was looking for what are they going to tell me. I was disappointed. Forget the green bar. Can you acknowledge me for what I did today? [61]

Prompts were reported as features in five studies [61, 65, 67, 71, 72]. Some participants reported certain prompts, like those to upgrade their applications, were annoying [65]. Gowin and colleagues [62] noted that participants who did not meet PA guidelines disliked prompts that reminded them to exercise because it caused guilt and they did not want to be bothered with exercise reminders, feeling they could decide for themselves when they wanted to exercise. Conversely, participants in Bentley and colleagues’ pilot study [72] noted participants asked for prompts in their application’s next iteration and, in their full study, participants valued prompts that supported engagement with the application, such as reminders to log data. Similar to prompts, feedback, often personalised in the form of step count, was also valued, but is discussed below as it refers to personalisation.

Goal setting via applications or wearables was another feature which was generally valued. Participants’ positive feelings and willingness to adopt it were noted in ten studies [61, 62, 64, 65, 67, 68, 70–73]. Goals in conjunction with feedback were credited with focusing participants:

It is very important for me to set goals… with a graphic representation, like a bar, for example, you have a guideline to exercise a specific amount of hours per week, then it would be very good to see, “oh right now I am in the red zone or the orange zone,” and when I am progressing, “I am in the green zone.” [67]

Several studies indicated people enjoyed elements of gamification [65], particularly in-application rewards [67] or competing to reach a coloured bar [61, 70], while others described the application or wearable itself as being like a game and enjoyed competing against themselves [59, 62, 64]. Mixed responses to rewards were noted by Middelweerd and colleagues [67], but what their participants did value was transparency in how rewards were calculated. As noted above, features like monitoring long-term progress [65, 67, 72] and ability to share data with healthcare professionals [65, 69] were also valued.

### The experience of personalised mHealth and physical activity

The value of personalisation was discussed in four of the included studies [64, 65, 69, 72], while four studies noted more personalised feedback would have been beneficial [61, 67, 71, 73]. In addition to personalisation, a desire to customise applications and to record additional individual-specific context was noted.

Feedback is a clear area where personalisation was evident and valued. Some delivered messages pushed by the application [61], while others relied on in-application statistics and trendlines to chart progress [72]. Participants in Bentley and colleagues’ study valued the accuracy provided by their system, noting that the feedback was “very truthful. It doesn’t hide or anything like that… It makes me know that I’m not reaching it and that I need to be doing physical activity” [72]. Many applications offered prompts to encourage PA or to encourage engagement with the application itself. This varied from automatic notifications [71] to maintaining a widget on the home screens of participants’ phones which provided a constant reminder of progress towards goals [72]. Applications and wearables frequently provided step count as a method for quantifying PA. Step count was unique to the individual and allowed them to monitor progress toward goals. A participant in Naslund, Aschbrenner and Bartels’ [64] study noted feedback in the form of step count provided something “tangible”, a proof of being active. Similarly, Knight and Petrella [69] noted that personal feedback allowed people to develop an understanding of where their PA level stood in relation to evidence-based guidelines. Participants noted personalised feedback increased engagement with the application [72] and supported motivation to reach their goals [64, 67, 71]:

Oh yeah, because it would send a message to my phone that says, “You’re only 650 steps away from your goal!” then I would go oh yeah, I could do that easy. And it’s 500 steps or 600 steps, I can do that. So I just added a few more walks back and forth to the laundry room; so it did encourage me meet and often exceed my step goal. [64]

Negative views on prompts providing feedback were noted in two studies [65, 67]. In contrast, two studies noted that while they were not always viewed as necessary, prompts were not perceived as irritating where participants were in full control of them [71] or where they could be disabled or amended to suit consumer preference [65].

Yeah [I haven't disabled the auditory alerts]. My running app will ping every so often… saying a friend has completed a run, or it's time for me to do a run, or something along those lines. [My application with a wristband device] sends me a little alert if I'm close to my goals, if I've got 2,000 more steps to go. [The auditory alert] doesn't really bother me. I just tune out. [65]

Fukuoka, Lindgren and Jong [61] noted their participants desired feedback that was more reflective of their effort. This study incorporated generic feedback in the form of messages which were viewed as less helpful:

[O]ne of the daily messages advised women to take a short walk before turning on the television. One subject reported: “I have two small children. I simply don’t have time to watch TV.”, another stated, “The daily message was not helpful for me, but the diary (was helpful). A half of the questions (for the daily message) were dumb. [61]

In addition to valuing personalised feedback, end-users reported wanting to customise their applications. Whether it was looking for additional self-monitoring capability or the ability to alter the application’s layout, three studies noted participants would have preferred to customise their applications [59, 65, 67]. With regards a running application, one participant suggested he would like to adjust what data the application collected: “I would love… to be able to record reps, and sets, and weights and things like that [if their running app were more customisable]” [65].

When discussing improving current applications, some users expressed interest in capturing additional context. Three studies noted the desire to record additional detail [67, 72, 73]. This included level of motivation and their satisfaction with a workout [67], and why they failed to complete a specific activity, with a view to identifying trends in their personal data [73]. Participants noted being able to capture additional context would have been valued:

[The system] doesn’t really take into account any outside factors. Like something [that the system doesn’t capture] happens and that’s why you’re in a bad mood… It’s just defined rules and if it doesn’t fall in that, then it doesn’t comply. [72]

### Technical and user issues in mHealth and their effect on experience

A variety of technical and user issues which impacted mHealth usage were noted across thirteen studies [59, 60, 62–68, 70–73]. As noted above, three studies alluded to an initial learning curve, with some participants finding mHealth components difficult to set up or use at first, though generally these issues were resolved through support [59, 64, 69].

Data entry, whether it was setting up an application or inputting PA data, was raised in seven studies, including a dislike for it [62, 67, 71, 73] or how easy it was to forget to log data [59, 68, 72]. It was described as “repetitive and tedious” and “monotonous and boring” by participants [73]. One participant reported using their application, “requires a lot of effort and I do not feel motivated enough to want to do it. Entering data was cumbersome” [73]. Similarly, concerns around battery life were raised in three studies [64, 67, 70].

These seemingly trivial issues are notable for their potential impact on application usage. Middelweerd and colleagues [67] noted concerns over application faults and battery or storage issues could be sufficient to trigger cessation of application use over time. Some participants reported challenges using the application itself. User issues included forgetting to use the application [67, 72] or forgetting to initiate PA monitoring features [67].

Six studies noted participants’ concerns around accuracy of recorded data or feedback [60, 63–65, 68, 72]. Though participants reported valuing “metrics” and “objective data” [72], the impact of inaccuracy was not explored in-depth, with only one study explicitly linking inaccuracy to mistrust [72]. However, if data were perceived as reasonably accurate, participants reported contentedness:

I went go-karting a while ago and [the app] thought I did like a hundred flights of stairs and thousands and thousands of steps in the hour I was driving around… I know it's never going to be exact, but if it's within a few hundred steps, then that's fine. [65]

Notably, the above inaccuracy overestimated PA in the participant’s favour.

Not all the technical issues experienced were insurmountable. Some participants relied on family members or co-workers to troubleshoot technical issues, whereas other participants attempted to mitigate the effects of inaccuracy independently: “I went kayaking and didn’t get any credit for movement in the kayak. So I put it (activity monitor) up to my (shirt) pocket (from waist) and then I did” [60].

In addition to practical considerations, the importance of an engaging and well-designed application, including the use of limited instructions, clearly understood language and basic numeracy, was noted in five studies [62, 66, 67, 72, 73]. Anderson, Burford and Emmerton [65] noted ease of use was key and that difficulty engaging with an application resulted in reduced usage. Similarly, Partridge and colleagues [66], who had incorporated a smartphone application into a larger intervention targeting weight loss, indicated that their participants reported concerns over design and navigation difficulties. As noted above, this prompted some to stop using the researchers’ applications and to revert to off-the-shelf alternatives that they had relied on prior to the study.

Data security and privacy were explored in one study and views were mixed [65]. For data like height and weight, participants had few concerns and were generally unconcerned with privacy: “I don't think about [data security], to be honest. This is going to sound terrible—maybe I'm just really naïve… I don't know, it doesn't really concern me. Probably, it should” [65]. However, when it came to third party access, e.g. health insurers, some participants raised concerns [65].

### Line-of-Argument synthesis

We found that the results of the studies were reciprocally translatable. Overall, the experiences of people using mHealth to support PA were, in the main, positive and many studies noted self-reported increased PA attributed to mHealth use. Across the included studies there was clear convergence on two categories of factors that influenced participants’ experiences of mHealth; personal factors and features of the device. Personal factors included prior experience and motivations for using mHealth. Features of the mHealth application were discussed extensively and included; personalisation, social features, feedback, prompts, goal setting, and gamification. These factors could work to support or hinder PA engagement through their influence on individual motivation. The other chief mechanism reported through which application use enabled PA was changes in self-awareness and strategising which facilitated PA.

The findings also reveal that the experience of mHealth use was not entirely unproblematic. Negative experiences of technical issues, navigation difficulty, data accuracy and security concerns, intrusion of prompts and notifications and challenges using the application were common. Other concerns noted were the potential for self-monitoring to cause subsequent anxiety and the demands of social comparison engendered by applications with social features.

## Discussion

The current meta-ethnography has systematically reviewed and synthesised the literature on the experiences of adults who have used mHealth applications to promote PA. While the focus of the original protocol was to explore the experience of using applications and applications and wearable activity monitors, a majority of the findings relate to applications and this was reflective of the included studies. The findings suggest that mHealth interventions for the promotion of PA, in the main, are perceived positively by the end-user. It highlights the role motivation plays in engaging in PA and how motivation is influenced through personal factors and through the device. Applications appeared to support strategising and problem solving, facilitating adaptation of routines to incorporate PA. Experiences of personalisation and self-monitoring were largely positive, although, important to note, is that self-monitoring can facilitate negative experiences in terms of frequent checking on progress and if goals go unmet. Notably, technical issues and poor design can also negatively influence experience.

One of the insights arising from the included studies was the influence of personal factors on the experience of mHealth. In some instances, limited prior mHealth experience led to a learning curve [64, 69], poorly designed applications were abandoned in favour of commercial applications [66] and individual motivations for application use differed, e.g. developing or maintaining existing behaviours [62]. In other instances, end-users reported a disinterest in using mHealth [59, 67, 69]. This observation dovetails with findings that personality traits may influence smartphone ownership and application preferences [75]. It also aligns with previous recommendations that PA interventions should consider minority group status and cultural competence, as well as age and the unique characteristics associated with each of those factors [76]. Indeed, most of included studies drew from adults in the Global North who were under the age of 65, so scope exists to further explore the experiences of other populations. These considerations begin to form the ‘digital divide’, a concept which includes socioeconomic status, age and geographic location, all of which can impact uptake of health interventions via smartphones [77]. Collectively, variation in personal factors may contribute to the mixed efficacy reported in previous evaluations of mHealth applications for the promotion of PA [26, 48] and should be considered when planning mHealth interventions.

Given the potential effect which personal factors may exert on the experience of using mHealth, it is unsurprising that personalisation was experienced positively by participants. Valued feedback was frequently tailored in response to recorded PA [72], whereas generic feedback was less valued [61]. The desire to customise the application itself, as well as the type of data collected, was also noted to be important to end-users [59, 60, 65, 67]. These results align with previous findings that tailoring can support engagement with interventions and improve PA outcomes [23, 24]. Thus, providing greater opportunities for personalisation and customisation in applications may offer some inoculation against disengagement.

Application developers incorporate a variety of features aimed at maintaining user engagement. In this review, features such as prompts, goal setting and gamification were all generally experienced positively. It has been suggested that strategies like prompting and push notifications may support habit formation [78]. Engagement strategies are critical as they may reduce attrition and increase exposure to the intervention, with one Internet-based PA promotion intervention involving adults with rheumatoid arthritis noting a dose-relationship between level of engagement and PA outcomes [79]. Application and design features that this review identified as valued by participants resonate with experts’ views on engagement strategies for an application to support reduced alcohol consumption, including ease of use, aesthetic design, feedback, tailored information, gamification, rewards, social comparison and connectivity, and prompts [80]. The importance of clear design, limited data entry and lack of technical issues were identified as supporting positive experiences of application use and supportive of PA. Technical issues related to difficulty operating the application have been implicated in attrition and cessation and have also been reported elsewhere [81]. This was exemplified by participants in Partridge and colleagues’ study [66] who disengaged from using the provided applications and reverted to applications used prior to the intervention.

The findings in relation to changes in thinking and features of the application that support engagement in PA reflect the growing body of literature on behaviour change. Michie and colleagues [82], in developing a taxonomy of behaviour change techniques for the promotion of PA, identified 93 distinct techniques. Several, specifically goal setting (behaviour), provision of rewards contingent on successful behaviour, prompting self-monitoring, provision of feedback on performance, facilitation of social comparison and social support, were noted in the included studies and appear across several third-order constructs. However, not all studies or applications contributed equally to all constructs or incorporated the same behaviour change strategies. This aligns with previous explorations of applications promoting PA which highlight both the variation and limited use of behaviour change techniques, with previous estimates of the number of techniques used ranging from one to twenty-one [83] and averaging between five [34] and 8.1 [84].

While behaviour change techniques were generally not reported in the included studies, inferences can be made about which techniques might be experienced positively. Comparing the current findings to Michie and colleagues’ behaviour change techniques for PA laid out in the CALO-RE taxonomy, overlap is evident. Firstly, goal setting, to increase specific behaviour, like step count, was noted in several studies [61, 62, 64, 65, 67, 68, 70–73]. Feedback was also noted to support motivation to reach goals [64, 67, 71]. Social support is noted in the literature as a unique behaviour change technique [82] and has been used effectively in community-based PA interventions [85]. Thus, it is unsurprising that application developers have capitalised on this and incorporated social media. However, this has been met with mixed results in the included studies. Similar results were noted by Dennison and colleagues [86] whilst exploring adults from the general population’s attitudes towards mHealth applications aimed at supporting behaviour change, including PA. Their participants also noted sharing to social media was unnecessary or off-putting, with the authors reporting that participants did not want to overtly appear weak or vulnerable. This aligns with the results of the included studies, particularly Anderson, Burford and Emmerton [65] wherein authors noted that participants were most interested in sharing notable achievements and highlights the importance of privacy. Thus, an additional challenge is how to incorporate social support in a meaningful way that is agreeable to users. Generally, good support for these behaviour change techniques was reported by participants. The facilitation of social comparison was met with mixed responses. However, even where it may have caused stress, it seemed likely to support PA. Of note, several studies prompted participants to self-monitor. While self-monitoring has been highlighted in the literature previously [87], participants in the included studies, in the main, disliked manually inputting data through applications. Thus, future mHealth interventions may seek to either automate data entry or make it easier, e.g. by building in ‘drop-down’ menus. Based on the perceptions of included participants, goal setting (to increase a particular behaviour) and providing feedback on performance may potentially be useful behaviour change techniques for future application developers, while behaviour change techniques like the facilitation of social comparison or self-monitoring might be adopted with caution.

In addition to resonating with behaviour change literature, the findings also reflect theories of motivation. Self-determination theory, which drills deeper into motivation by differentiating between intrinsic and extrinsic types, has previously been applied in the context of supporting interventions for promoting PA [88]. Considering how theories of motivation might augment behaviour change techniques may be useful in future research.

The findings noted in the current study resonate well with findings from a qualitative study by Casey and colleagues [89] exploring the experiences of primary healthcare users using mHealth to promote PA. This study was excluded from the current systematic review and meta-synthesis as the inclusion of participants under 18 years rendered that it did not meet our age-based inclusion criteria. In line with the findings of this review, Casey and colleagues [89] found that both personal factors and features of the device influenced motivation and engagement in PA. Negative experiences included frustration when goals were unmet and where technical and user issues were noted, including concerns over battery life and forgetting to bring their phones as they exercised.

Of note, in exploring end-users’ perspectives of mHealth for promotion of PA, little focus was given to data security and privacy. This is noteworthy given the potentially sensitive information collected by the applications, particularly as it pertains to users’ health and condition, and because many of the applications in the included studies were commercial products, arguably resulting in end-users or researchers feeling they have less control over how their data is stored. Lupton [42], having devoted significant attention to the concept of self-monitoring, has noted that though concern over data storage practices seems to be rising, many are unaware of what happens to their personal data once it is transferred to cloud archives and what measures can be taken to protect it, which resonates with findings noted above by Anderson, Burford and Emmerton [65]. With continued commodification of personal data [90], the limited control by end-users over third party access to data and also data breaches [91], particularly the 2018 data breach of Under Armour’s PA and diet application ‘MyFitnessPal’, data protection and issues of privacy will likely become more pressing concerns for application design going forward.

Across the identified themes, increased PA was aspired to among participants and the belief that individual motivation drives PA permeated the findings. The role of individual motivation in maintaining health aligns with neoliberal policy and has been critiqued [90]. A focus on self-monitoring shifts responsibility for health outcomes to the individual while removing focus from the influence of the wider socio-political environment in which the individual operates. Throughout the findings, there was limited attention given to the wider contextual factors shaping PA, such as the environment and the policy and legislative context. Environment [92], sedentary occupations [93] or temporal factors, like rotating seasons which can influence occupational or recreational patterns, as noted in Eisenhauer and colleagues’ [60] study, all exert influence on PA. Feelings of shame and guilt as a result of self-monitoring or, conversely, positive experiences of feedback on increased PA are consequences of the belief that the individual is wholly responsible for their level of PA. Arguably, applications are frequently limited in their focus and have difficulty addressing the wider social determinants of health generally and PA specifically [90]. While applications may support individuals in making changes to their PA levels, it is important to remain cognisant of the expectations that mHealth can place on individuals and to acknowledge the influences of factors beyond the individual’s control.

### Strengths and limitations

The current meta-ethnography was the first to complete a systematic review and qualitative synthesis of the existing literature in the area of mHealth and PA promotion. Nevertheless, these findings should be interpreted within the context of the study’s limitations. Meta-ethnography, by design, is interpretive rather than aggregative and other researchers may have drawn different conclusions from the data. However, to add rigour to the current study, a numerical approach was taken and the number of studies contributing to each third-order construct noted. Effort was made to illustrate each construct through quotations and contradictory data was highlighted. This required considerable time and prolonged engagement with the data. Reflexivity refers in part to the way in which the researcher and research design influence findings [94]. It was felt that the researchers’ experience provided good counterbalance. KR’s background was in qualitative research, while SH came from a background in quantitative research. DC, an early-career doctoral researcher, however, had undertaken a postgraduate-level course in qualitative research methods whilst completing the analysis. It was felt that their differing insights both resulted in valued and unique contributions to the analysis of third-order constructs.

Several limitations affect the direct generalisability of the current findings. Firstly, samples are drawn from a mixture of healthy, community-dwelling adults and patient populations. Secondly, a range of ages is included. Prior literature has argued that different generations vary in the expectations they have from technology. “Digital natives”, those born after 1980 and who have grown up immersed in technology from a young age, differ from “digital immigrants”, those born before 1980 who adopted technology later in life [95]. Though technology adoption in older adults is on the rise, it continues to lag the general population [96] and has been attributed to lack of confidence, interest or skills [97]. Younger participants may be more adept at using smartphones and wearables and thus more receptive to mHealth interventions and age likely influences how mHealth is experienced. Thirdly, a key element of qualitative research is defining the sample universe. This requires making explicit a study sample’s defining characteristics with a view to making generalisations from findings valid and transparent [98]. A finding noted in Table 1 was that participants’ prior mHealth experience was inconsistently reported across studies. Prior research has indicated adoption of health technology is influenced by familiarity, particularly in older adults [99, 100]. Thus, by more explicitly defining sample characteristics the validity of generalisations drawn from some of the included studies could have been improved [98]. Finally, there exists risk of response and self-selection bias. Seven of the included studies used applications developed or customised by the researchers [59, 61, 66, 68, 70–72]. As Årsand and colleagues [70] noted, their participants may have been inclined to offer more favourable feedback having been heavily involved in the application’s development, resulting in response bias. Self-selection bias, where individuals who have consented to take part in the included studies differs from those who did not in ways unrelated to inclusion criteria, is unavoidable [98]. It remains worth noting, however, that those recruited for the included studies may have been pre-disposed to both or either using mHealth components or increasing their PA. Thus, variation in sample characteristics, age, as well asresponse and self-selection bias may have exerted influence and must be considered when interpreting the findings of the current review. However, with these limitations in mind, the current study’s key strength is that it offers insight into the experience of using mHealth applications for PA promotion and serves to highlight that further research in the area is warranted.

### Implications

The third-order constructs generated above form a foundation for understanding the experiences of adults using mHealth applications to promote PA. However, given that a variety of health statuses were represented amongst participants, future research should explore whether these findings reflect the experiences of specific populations targeted for promotion of PA. Further, as experiences of mHealth generally appeared positive in the face of mixed efficacy reported by several reviews, further exploration is warranted to identify ways of overcoming barriers to motivation and engagement. Given that the current study aims to inform the future design of a complex health intervention aimed at promoting PA in people with stroke, further research is warranted to explore their unique needs.

Additionally, qualitative research and participant experience offer only a partial account of the role mHealth can play in the promotion of PA. The findings of this review should be triangulated with research that investigates whether user experiences are associated with particular behaviour change techniques or with objectively measured changes in PA. By adopting a triangulation approach, the findings of the current study can be strengthened [101] and more effective mHealth interventions aimed at the promotion of PA can be developed.

The current review also highlights the issue of rigour in qualitative research in this area. The CASP tool indicated limited consideration of the relationship between researcher and participants across the included studies. This is notable as the findings from qualitative research are considered a joint product of this relationship and, by examining it, the researcher is afforded an opportunity to demonstrate reflexivity which can lend integrity and trustworthiness to their findings [102]. Future qualitative research exploring mHealth and PA promotion could benefit from following formal guidelines, like the Consolidated Criteria for Reporting Qualitative Research which emphasises reporting on reflexivity to improve rigour and the conduct of qualitative research [103].

In terms of implications for healthcare providers, the current study highlights the importance of considering the practicalities of implementing mHealth-based interventions. Naslund, Aschbrenner and Bartels [64] suggested that their participants were disadvantaged as they had limited prior experience with smartphones which lead to an initial learning curve at the start of their mHealth intervention. Arguably, individuals with chronic conditions may have less disposable income and may be older, both of which may reduce the likelihood of smartphone ownership [32]. This was remedied, in the study by Naslund and colleagues [64], through provision of mHealth components at no cost to the participants and through patient education but illustrates the requirement that interventions be tailored to their intended recipients. Additionally, with regards selection of applications in clinical practice, healthcare practitioners and consumers of applications generally might consider utilising applications which offer features that have been found agreeable to end-users, e.g. the ability to self-monitor, set goals and receive feedback. Finally, the level of tailoring the application offers and the overarching goals of the user, whether it be short or long-term use, should also be given consideration.

### Conclusion

The current findings highlight the role which personal factors and mHealth application features play in facilitating changes in thinking, including awareness, strategising and motivation, to support increased PA. Adverse effects were reported as a result of self-monitoring as well as technical and user issues. The identified themes highlight challenges for future research to target, with a view to maximising positive experiences and ensuring negative experiences are mitigated, during the promotion of PA through mHealth.

## Supporting information

S1 File ENTREQ checklist. (DOCX)

S2 File MEDLINE search terms. (DOCX)

S3 File PRISMA checklist. (DOCX)

S1 Table Quality appraisal. (XLSX)

## References

[1] Alves AJ, Viana JL, Cavalcante SL, Oliveira NL, Duarte JA, Mota J, et al Physical activity in primary and secondary prevention of cardiovascular disease: Overview updated. World journal of cardiology. 2016;8(10):575 10.4330/wjc.v8.i10.575 27847558 10.4330/wjc.v8.i10.575 PMC5088363

[2] World Health Organization. Global recommendations on physical activity for health Geneva, Switzerland: World Health Organization; 2010 [22nd June 2018]. Available from: http://www.who.int/dietphysicalactivity/publications/9789241599979/en/.

[3] Zhang D, Liu X, Liu Y, Sun X, Wang B, Ren Y, et al Leisure-time physical activity and incident metabolic syndrome: a systematic review and dose-response meta-analysis of cohort studies. Metabolism-Clinical and Experimental. 2017;75:36–44. 10.1016/j.metabol.2017.08.001 28927737 10.1016/j.metabol.2017.08.001

[4] Lear SA, Hu W, Rangarajan S, Gasevic D, Leong D, Iqbal R, et al The effect of physical activity on mortality and cardiovascular disease in 130 000 people from 17 high-income, middle-income, and low-income countries: the PURE study. The Lancet. 2017;390(10113):2643–54. 10.1016/S0140-6736(17)31634-3 28943267

[5] Saint‐Maurice PF, Troiano RP, Matthews CE, Kraus WE. Moderate‐to‐Vigorous Physical Activity and All‐Cause Mortality: Do Bouts Matter? Journal of the American Heart Association. 2018;7(6):e007678 10.1161/JAHA.117.007678 29567764 10.1161/JAHA.117.007678 PMC5907548

[6] Ding D, Lawson KD, Kolbe-Alexander TL, Finkelstein EA, Katzmarzyk PT, Van Mechelen W, et al The economic burden of physical inactivity: a global analysis of major non-communicable diseases. The Lancet. 2016;388(10051):1311–24. 10.1016/S0140-6736(16)30383-X 27475266

[7] World Health Organization. Global action plan on physical activity 2018–2030: more active people for a healthier world Geneva, Switzerland: World Health Organization; 2018 [22nd June 2018]. Available from: http://apps.who.int/iris/bitstream/handle/10665/272722/9789241514187-eng.pdf.

[8] Bull F, Biddle S, Buchner D, Ferguson R, Foster C, Fox K. Physical activity guidelines in the UK: review and recommendations School of Sport, Exercise and Health Sciences, Loughborough University 2010.

[9] Tremblay MS, Warburton DE, Janssen I, Paterson DH, Latimer AE, Rhodes RE, et al New Canadian physical activity guidelines. Applied Physiology, Nutrition, and Metabolism. 2011;36(1):36–46. 10.1139/H11-009 21326376 10.1139/H11-009

[10] US Department of Health and Human Services. 2008 physical activity guidelines for Americans Washington, D.C.: US Department of Health and Human Services; 2008 [22nd June 2018]. Available from: https://health.gov/paguidelines/guidelines/default.aspx.

[11] Foundation Stroke. Clinical Guidelines for Stroke Management 2017. Melbourne, Australia: National Stroke Foundation; 2017.

[12] Wein T, Lindsay MP, Côté R, Foley N, Berlingieri J, Bhogal S, et al Canadian stroke best practice recommendations: Secondary prevention of stroke, practice guidelines, update 2017. International Journal of Stroke.1747493017743062. 10.1177/1747493017743062 29171361

[13] Lee I-M, Shiroma EJ, Lobelo F, Puska P, Blair SN, Katzmarzyk PT, et al Effect of physical inactivity on major non-communicable diseases worldwide: an analysis of burden of disease and life expectancy. The Lancet. 2012;380(9838):219–29. 10.1016/S0140-6736(12)61031-9 PMC3645500 22818936

[14] Jackson S, Mercer C, Singer BJ. An exploration of factors influencing physical activity levels amongst a cohort of people living in the community after stroke in the south of England. Disability and rehabilitation. 2018;40(4):414–24. 10.1080/09638288.2016.1258437 28029070 10.1080/09638288.2016.1258437

[15] O'Donnell MJ, Chin SL, Rangarajan S, Xavier D, Liu L, Zhang H, et al Global and regional effects of potentially modifiable risk factors associated with acute stroke in 32 countries (INTERSTROKE): a case-control study. The Lancet. 2016;388(10046):761–75. 10.1016/S0140-6736(16)30506-2 27431356

[16] Hardie K, Hankey GJ, Jamrozik K, Broadhurst RJ, Anderson C. Ten-year risk of first recurrent stroke and disability after first-ever stroke in the Perth Community Stroke Study. Stroke. 2004;35(3):731–5. 10.1161/01.STR.0000116183.50167.D9 14764929 10.1161/01.STR.0000116183.50167.D9

[17] Saunders DH, Sanderson M, Hayes S, Kilrane M, Greig CA, Brazzelli M, et al Physical fitness training for stroke patients. The Cochrane Library. 2016. 10.1002/14651858.CD003316.pub6 PMC6464717 27010219

[18] Field MJ, Gebruers N, Shanmuga Sundaram T, Nicholson S, Mead G. Physical activity after stroke: a systematic review and meta-analysis. ISRN Stroke. 2013;2013.

[19] Tudor-Locke C, Craig CL, Brown WJ, Clemes SA, De Cocker K, Giles-Corti B, et al How many steps/day are enough? For adults. The International Journal of Behavioral Nutrition and Physical Activity. 2011;8. 2012-31409-001. 10.1186/1479-5868-8-79 PMC3197470 21798015

[20] Tudor-Locke C, Craig CL, Aoyagi Y, Bell RC, Croteau KA, De Bourdeaudhuij I, et al How many steps/day are enough? For older adults and special populations. International Journal of Behavioral Nutrition and Physical Activity. 2011;8(1):80. 21798044 10.1186/1479-5868-8-80 PMC3169444

[21] Kreuter MW, Bull FC, Clark EM, Oswald DL. Understanding how people process health information: a comparison of tailored and nontailored weight-loss materials. Health Psychology. 1999;18(5):487 10519465 10.1037//0278-6133.18.5.487

[22] Rimer BK, Kreuter MW. Advancing tailored health communication: A persuasion and message effects perspective. Journal of communication. 2006;56:S184–S201.

[23] Short CE, James EL, Plotnikoff RC, Girgis A. Efficacy of tailored-print interventions to promote physical activity: a systematic review of randomised trials. International Journal of Behavioral Nutrition and Physical Activity. 2011;8(1):113. 21999329 10.1186/1479-5868-8-113 PMC3214130

[24] Davies CA, Spence JC, Vandelanotte C, Caperchione CM, Mummery WK. Meta-analysis of internet-delivered interventions to increase physical activity levels. International Journal of Behavioral Nutrition and Physical Activity. 2012;9(1):52. 22546283 10.1186/1479-5868-9-52 PMC3464872

[25] Schoeppe S, Alley S, Van Lippevelde W, Bray NA, Williams SL, Duncan MJ, et al Efficacy of interventions that use apps to improve diet, physical activity and sedentary behaviour: a systematic review. International Journal of Behavioral Nutrition and Physical Activity. 2016;13(1):127 10.1186/s12966-016-0454-y 27927218 10.1186/s12966-016-0454-y PMC5142356

[26] Stuckey MI, Carter SW, Knight E. The role of smartphones in encouraging physical activity in adults. International journal of general medicine. 2017;10:293 10.2147/IJGM.S134095 28979157 10.2147/IJGM.S134095 PMC5602432

[27] Nahum-Shani I, Smith SN, Spring BJ, Collins LM, Witkiewitz K, Tewari A, et al Just-in-time adaptive interventions (JITAIs) in mobile health: key components and design principles for ongoing health behavior support. Annals of Behavioral Medicine. 2017;52(6):446–62. 10.1007/s12160-016-9830-8 PMC5364076 27663578

[28] Dijkhuis TB, Blaauw FJ, van Ittersum MW, Velthuijsen H, Aiello M. Personalized physical activity coaching: a machine learning approach. Sensors. 2018;18(2):623. 10.3390/s18020623 PMC5856112 29463052

[29] Prince SA, Adamo KB, Hamel ME, Hardt J, Gorber SC, Tremblay M. A comparison of direct versus self-report measures for assessing physical activity in adults: a systematic review. International Journal of Behavioral Nutrition and Physical Activity. 2008;5(1):56. 18990237 10.1186/1479-5868-5-56 PMC2588639

[30] Skender S, Ose J, Chang-Claude J, Paskow M, Brühmann B, Siegel EM, et al Accelerometry and physical activity questionnaires-a systematic review. BMC public health. 2016;16(1):515. 27306667 10.1186/s12889-016-3172-0 PMC4910242

[31] Agarwal S, LeFevre AE, Lee J, L’Engle K, Mehl G, Sinha C, et al Guidelines for reporting of health interventions using mobile phones: mobile health (mHealth) evidence reporting and assessment (mERA) checklist. BMJ. 2016;352:i1174 10.1136/bmj.i1174 26988021 10.1136/bmj.i1174

[32] Poushter J. Smartphone ownership and internet usage continues to climb in emerging economies. Pew Research Center. 2016;22.

[33] Conroy DE, Yang C-H, Maher JP. Behavior change techniques in top-ranked mobile apps for physical activity. American journal of preventive medicine. 2014;46(6):649–52. 10.1016/j.amepre.2014.01.010 24842742 10.1016/j.amepre.2014.01.010

[34] Middelweerd A, Mollee JS, van der Wal CN, Brug J, te Velde SJ. Apps to promote physical activity among adults: A review and content analysis. The International Journal of Behavioral Nutrition and Physical Activity. 2014;11. 2014-40717-001. 10.1186/s12966-014-0097-9 PMC4132213 25059981

[35] Hoj TH, Covey EL, Jones AC, Haines AC, Hall PC, Crookston BT, et al How Do Apps Work? An Analysis of Physical Activity App Users’ Perceptions of Behavior Change Mechanisms. JMIR mHealth and uHealth. 2017;5(8). 10.2196/mhealth.7206 PMC5561388 28778846

[36] Miyamoto SW, Henderson S, Young HM, Pande A, Han JJ. Tracking health data is not enough: a qualitative exploration of the role of healthcare partnerships and mHealth technology to promote physical activity and to sustain behavior change. JMIR mHealth and uHealth. 2016;4(1). 10.2196/mhealth.4814 PMC4758807 26792225

[37] Peiris D, Miranda JJ, Mohr DC. Going beyond killer apps: building a better mHealth evidence base. BMJ Specialist Journals; 2018. 10.1136/bmjgh-2017-000676 PMC5841531 29527353

[38] Facey K, Boivin A, Gracia J, Hansen HP, Scalzo AL, Mossman J, et al Patients' perspectives in health technology assessment: a route to robust evidence and fair deliberation. International journal of technology assessment in health care. 2010;26(3):334–40. 10.1017/S0266462310000395 20584364 10.1017/S0266462310000395

[39] O’Connor S, Hanlon P, O’Donnell CA, Garcia S, Glanville J, Mair FS. Understanding factors affecting patient and public engagement and recruitment to digital health interventions: a systematic review of qualitative studies. BMC medical informatics and decision making. 2016;16(1):120 10.1186/s12911-016-0359-3 27630020 10.1186/s12911-016-0359-3 PMC5024516

[40] Sharon T. Self-tracking for health and the quantified self: Re-articulating autonomy, solidarity, and authenticity in an age of personalized healthcare. Philosophy & Technology. 2017;30(1):93–121.

[41] Lupton D. Quantifying the body: monitoring and measuring health in the age of mHealth technologies. Critical Public Health. 2013;23(4):393–403.

[42] Lupton D. Personal data practices in the age of lively data. Digital Sociologies. 2016:335–50.

[43] Knowles SE, Toms G, Sanders C, Bee P, Lovell K, Rennick-Egglestone S, et al Qualitative meta-synthesis of user experience of computerised therapy for depression and anxiety. PLOS ONE. 2014;9(1):e84323 10.1371/journal.pone.0084323 24465404 10.1371/journal.pone.0084323 PMC3894944

[44] Brunton L, Bower P, Sanders C. The contradictions of telehealth user experience in chronic obstructive pulmonary disease (COPD): a qualitative meta-synthesis. PLoS One. 2015;10(10):e0139561 10.1371/journal.pone.0139561 26465333 10.1371/journal.pone.0139561 PMC4605508

[45] Pearce G, Pinnock H, Epiphaniou E, Parke HL, Heavey E, Griffiths CJ, et al Experiences of self-management support following a stroke: a meta-review of qualitative systematic reviews. PloS one. 2015;10(12):e0141803 10.1371/journal.pone.0141803 26657458 10.1371/journal.pone.0141803 PMC4682853

[46] Craig P, Dieppe P, Macintyre S, Michie S, Nazareth I, Petticrew M. Developing and evaluating complex interventions: the new Medical Research Council guidance. International journal of nursing studies. 2013;50(5):587–92. 10.1016/j.ijnurstu.2012.09.010 23159157 10.1016/j.ijnurstu.2012.09.010

[47] Coorey GM, Neubeck L, Mulley J, Redfern J. Effectiveness, acceptability and usefulness of mobile applications for cardiovascular disease self-management: Systematic review with meta-synthesis of quantitative and qualitative data. European journal of preventive cardiology. 2018:2047487317750913. 10.1177/2047487317750913 29313363

[48] Coughlin SS, Whitehead M, Sheats JQ, Mastromonico J, Smith S. A review of smartphone applications for promoting physical activity. Jacobs journal of community medicine. 2016;2(1). PMC4811195 27034992

[49] Noblit GW, Hare RD. Meta-ethnography: Synthesizing qualitative studies: sage; 1988.

[50] Barnett-Page E, Thomas J. Methods for the synthesis of qualitative research: a critical review. BMC Medical Research Methodology. 2009;9(1):59. 19671152 10.1186/1471-2288-9-59 PMC3224695

[51] Tong A, McInnes E, Craig J, Flemming K, Oliver S. Enhancing transparency in reporting the synthesis of qualitative research: ENTREQ. BMC medical research methodology. 2012;12(1):181. 23185978 10.1186/1471-2288-12-181 PMC3552766

[52] DeJean D, Giacomini M, Simeonov D, Smith A. Finding qualitative research evidence for health technology assessment. Qualitative health research. 2016;26(10):1307–17. 10.1177/1049732316644429 27117960 10.1177/1049732316644429

[53] Daly LM, Horey D, Middleton PF, Boyle FM, Flenady V. The effect of mobile application interventions on influencing healthy maternal behaviour and improving perinatal health outcomes: a systematic review protocol. Systematic reviews. 2017;6(1):26 10.1186/s13643-017-0424-8 28179012 10.1186/s13643-017-0424-8 PMC5299644

[54] Critical Appraisal Skills Programme. CASP Qualitative Checklist: Critical Appraisal Skills Programme; 2018. Available from: https://casp-uk.net/casp-tools-checklists/.

[55] Hannes K, Lockwood C, Pearson A. A comparative analysis of three online appraisal instruments’ ability to assess validity in qualitative research. Qualitative health research. 2010;20(12):1736–43. 10.1177/1049732310378656 20671302 10.1177/1049732310378656

[56] Ciliska D, Thomas H, Buffett C. A compendium of critical appraisal tools for public health practice. links. 2008.

[57] Hannes K, Lockwood C. Synthesizing qualitative research: choosing the right approach: John Wiley & Sons; 2011.

[58] Saini M, Shlonsky A. Systematic synthesis of qualitative research: OUP USA; 2012.

[59] Buman MP, Epstein DR, Gutierrez M, Herb C, Hollingshead K, Huberty JL, et al BeWell24: Development and process evaluation of a smartphone 'app' to improve sleep, sedentary, and active behaviors in US veterans with increased metabolic risk. Translational Behavioral Medicine. 2016;6(3):438–48. 10.1007/s13142-015-0359-3 2016-40180-012. 27528532 10.1007/s13142-015-0359-3 PMC4987607

[60] Eisenhauer CM, Hageman PA, Rowland S, Becker BJ, Barnason SA, Pullen CH. Acceptability of mHealth Technology for Self-Monitoring Eating and Activity among Rural Men. Public Health Nursing (Boston, Mass). 2017;34(2):138–46. 10.1111/phn.12297 . 27757986 10.1111/phn.12297

[61] Fukuoka Y, Lindgren T, Jong S. Qualitative exploration of the acceptability of a mobile phone and pedometer-based physical activity program in a diverse sample of sedentary women. Public Health Nursing (Boston, Mass). 2012;29(3):232–40. 10.1111/j.1525-1446.2011.00997.x . 22512424 10.1111/j.1525-1446.2011.00997.x PMC4219361

[62] Gowin M, Cheney M, Gwin S, Franklin Wann T. Health and Fitness App Use in College Students: A Qualitative Study. American Journal of Health Education. 2015;46(4):223–30. 10.1080/19325037.2015.1044140 . Language: English. Entry Date: 20150714. Revision Date: 20150923. Publication Type: Journal Article.

[63] Lewis ZH, Ottenbacher KJ, Fisher SR, Jennings K, Brown AF, Swartz MC, et al The feasibility and RE-AIM evaluation of the TAME health pilot study. International Journal of Behavioral Nutrition & Physical Activity. 2017;14:1–15. 10.1186/s12966-017-0560-5 . Language: English. Entry Date: 20170821. Revision Date: 20170822. Publication Type: Article. Journal Subset: Allied Health. 28807041 10.1186/s12966-017-0560-5 PMC5556663

[64] Naslund JA, Aschbrenner KA, Bartels SJ. Wearable devices and smartphones for activity tracking among people with serious mental illness. Mental Health and Physical Activity. 2016;10:10–7. 10.1016/j.mhpa.2016.02.001 2016-29820-004. 27134654 10.1016/j.mhpa.2016.02.001 PMC4845759

[65] Anderson K, Burford O, Emmerton L. Mobile Health Apps to Facilitate Self-Care: A Qualitative Study of User Experiences. Plos One. 2016;11(5):e0156164-e. 10.1371/journal.pone.0156164 . 27214203 10.1371/journal.pone.0156164 PMC4876999

[66] Partridge SR, Allman-Farinelli M, McGeechan K, Balestracci K, Wong ATY, Hebden L, et al Process evaluation of TXT2BFiT: A multi-component mHealth randomised controlled trial to prevent weight gain in young adults. The International Journal of Behavioral Nutrition and Physical Activity. 2016;13. 2016-03672-001. 10.1186/s12966-016-0329-2 PMC4717560 26785637

[67] Middelweerd A, van der Laan DM, van Stralen MM, Mollee JS, Stuij M, te Velde SJ, et al What features do Dutch university students prefer in a smartphone application for promotion of physical activity? A qualitative approach. The International Journal of Behavioral Nutrition and Physical Activity. 2015;12. 2015-11968-001. 10.1186/s12966-015-0189-1 PMC4359580 25889577

[68] van der Weegen S, Verwey R, Tange HJ, Spreeuwenberg MD, de Witte LP. Usability testing of a monitoring and feedback tool to stimulate physical activity. Patient preference and adherence. 2014;8:311 10.2147/PPA.S57961 24669188 10.2147/PPA.S57961 PMC3962312

[69] Knight E, Petrella RJ. Prescribing physical activity for healthy aging: longitudinal follow-up and mixed method analysis of a primary care intervention. The Physician And Sportsmedicine. 2014;42(4):30–8. 10.3810/psm.2014.11.2089 . 25419886 10.3810/psm.2014.11.2089

[70] Årsand E, Tatara N, Østengen G, Hartvigsen G. Mobile phone-based self-management tools for type 2 diabetes: the few touch application. Journal Of Diabetes Science And Technology. 2010;4(2):328–36. 10.1177/193229681000400213 . 20307393 10.1177/193229681000400213 PMC2864168

[71] Morrison LG, Hargood C, Lin SX, Dennison L, Joseph J, Hughes S, et al Understanding usage of a hybrid website and smartphone app for weight management: a mixed-methods study. Journal Of Medical Internet Research. 2014;16(10):e201–e. 10.2196/jmir.3579 . 25355131 10.2196/jmir.3579 PMC4259922

[72] Bentley F, Tollmar K, Stephenson P, Levy L, Jones B, Robertson S, et al Health mashups: Presenting statistical patterns between wellbeing data and context in natural language to promote behavior change. ACM Transactions on Computer-Human Interaction. 2013;20(5):1–27. 10.1145/2503823 2014-01620-004.

[73] Ahtinen A, Isomursu M, Ramiah S, Blom J. Advise, acknowledge, grow and engage: Design principles for a mobile wellness application to support physical activity. International Journal of Mobile Human Computer Interaction. 2013;5(4):20–55. 10.4018/ijmhci.2013100102 2014-20292-002.

[74] Dixon-Woods M, Sutton A, Shaw R, Miller T, Smith J, Young B, et al Appraising qualitative research for inclusion in systematic reviews: a quantitative and qualitative comparison of three methods. Journal of health services research & policy. 2007;12(1):42–7. 17244397 10.1258/135581907779497486

[75] Lane W, Manner C. The impact of personality traits on smartphone ownership and use. International Journal of Business and Social Science. 2011;2(17).

[76] Lewis BA, Napolitano MA, Buman MP, Williams DM, Nigg CR. Future directions in physical activity intervention research: expanding our focus to sedentary behaviors, technology, and dissemination. Journal of behavioral medicine. 2017;40(1):112–26. 10.1007/s10865-016-9797-8 27722907 10.1007/s10865-016-9797-8 PMC5296224

[77] Bert F, Giacometti M, Gualano MR, Siliquini R. Smartphones and health promotion: a review of the evidence. Journal of medical systems. 2014;38(1):9995 10.1007/s10916-013-9995-7 24346929 10.1007/s10916-013-9995-7

[78] Fry JP, Neff RA. Periodic prompts and reminders in health promotion and health behavior interventions: systematic review. Journal of medical Internet research. 2009;11(2). 10.2196/jmir.1138 PMC2762806 19632970

[79] Van den Berg M, Ronday H, Peeters A, Voogt-van Der Harst E, Munneke M, Breedveld F, et al Engagement and satisfaction with an Internet-based physical activity intervention in patients with rheumatoid arthritis. Rheumatology. 2006;46(3):545–52. 10.1093/rheumatology/kel341 17043050 10.1093/rheumatology/kel341

[80] Garnett C, Crane D, West R, Brown J, Michie S. Identification of behavior change techniques and engagement strategies to design a smartphone app to reduce alcohol consumption using a formal consensus method. JMIR mHealth and uHealth. 2015;3(2). 10.2196/mhealth.3895 PMC4526967 26123578

[81] Gilson ND, Pavey TG, Vandelanotte C, Duncan MJ, Gomersall SR, Trost SG, et al Chronic disease risks and use of a smartphone application during a physical activity and dietary intervention in Australian truck drivers. Australian & New Zealand Journal of Public Health. 2016;40(1):91–3. 10.1111/1753-6405.12501 . Language: English. Entry Date: 20160204. Revision Date: 20170131. Publication Type: Article. Journal Subset: Australia & New Zealand. 26713400 10.1111/1753-6405.12501

[82] Michie S, Richardson M, Johnston M, Abraham C, Francis J, Hardeman W, et al The behavior change technique taxonomy (v1) of 93 hierarchically clustered techniques: building an international consensus for the reporting of behavior change interventions. Annals of behavioral medicine. 2013;46(1):81–95. 10.1007/s12160-013-9486-6 23512568 10.1007/s12160-013-9486-6

[83] Yang C-H, Maher JP, Conroy DE. Implementation of behavior change techniques in mobile applications for physical activity. American journal of preventive medicine. 2015;48(4):452–5. 10.1016/j.amepre.2014.10.010 25576494 10.1016/j.amepre.2014.10.010

[84] Direito A, Dale LP, Shields E, Dobson R, Whittaker R, Maddison R. Do physical activity and dietary smartphone applications incorporate evidence-based behaviour change techniques? BMC public health. 2014;14(1):646. 24965805 10.1186/1471-2458-14-646 PMC4080693

[85] Heath GW, Parra DC, Sarmiento OL, Andersen LB, Owen N, Goenka S, et al Evidence-based intervention in physical activity: lessons from around the world. The lancet. 2012;380(9838):272–81. 10.1016/S0140-6736(12)60816-2 PMC4978123 22818939

[86] Dennison L, Morrison L, Conway G, Yardley L. Opportunities and challenges for smartphone applications in supporting health behavior change: qualitative study. Journal of medical Internet research. 2013;15(4). 10.2196/jmir.2583 PMC3636318 23598614

[87] Michie S, Ashford S, Sniehotta FF, Dombrowski SU, Bishop A, French DP. A refined taxonomy of behaviour change techniques to help people change their physical activity and healthy eating behaviours: the CALO-RE taxonomy. Psychology & Health. 2011;26(11):1479–98. 21678185 10.1080/08870446.2010.540664

[88] Fortier MS, Duda JL, Guerin E, Teixeira PJ. Promoting physical activity: development and testing of self-determination theory-based interventions. International Journal of Behavioral Nutrition and Physical Activity. 2012;9(1):20. 22385751 10.1186/1479-5868-9-20 PMC3353256

[89] Casey M, Hayes PS, Glynn F, Olaighin Gi, Heaney D, Murphy AW, et al Patients' experiences of using a smartphone application to increase physical activity: the SMART MOVE qualitative study in primary care. The British Journal Of General Practice: The Journal Of The Royal College Of General Practitioners. 2014;64(625):e500–e8. 10.3399/bjgp14X680989 . 25071063 10.3399/bjgp14X680989 PMC4111343

[90] Lupton D. Health promotion in the digital era: a critical commentary. Health Promotion International. 2014;30(1):174–83. 10.1093/heapro/dau091 25320120 10.1093/heapro/dau091

[91] Glenn T, Monteith S. Privacy in the digital world: medical and health data outside of HIPAA protections. Current psychiatry reports. 2014;16(11):494 10.1007/s11920-014-0494-4 25218603 10.1007/s11920-014-0494-4

[92] Parks S, Housemann RA, Brownson RC. Differential correlates of physical activity in urban and rural adults of various socioeconomic backgrounds in the United States. Journal of Epidemiology & Community Health. 2003;57(1):29–35. 12490645 10.1136/jech.57.1.29 PMC1732269

[93] Clark B, Sugiyama T. Prevalence, trends, and correlates of sedentary behavior Physical Activity, Exercise, Sedentary Behavior and Health: Springer; 2015 p. 79–90.

[94] Finlay L. Negotiating the swamp: the opportunity and challenge of reflexivity in research practice. Qualitative research. 2002;2(2):209–30.

[95] Prensky M. Digital natives, digital immigrants part 1. On the horizon. 2001;9(5):1–6.

[96] Anderson M, Perrin A. Tech adoption climbs among older adults. Pew Research Center 2017:1–22.

[97] Siren A, Knudsen SG. Older adults and emerging digital service delivery: A mixed methods study on information and communications technology use, skills, and attitudes. Journal of aging & social policy. 2017;29(1):35–50. 27215167 10.1080/08959420.2016.1187036

[98] Robinson OC. Sampling in interview-based qualitative research: A theoretical and practical guide. Qualitative research in psychology. 2014;11(1):25–41.

[99] Fischer SH, David D, Crotty BH, Dierks M, Safran C. Acceptance and use of health information technology by community-dwelling elders. International journal of medical informatics. 2014;83(9):624–35. 10.1016/j.ijmedinf.2014.06.005 24996581 10.1016/j.ijmedinf.2014.06.005 PMC4144164

[100] Peek ST, Wouters EJ, van Hoof J, Luijkx KG, Boeije HR, Vrijhoef HJ. Factors influencing acceptance of technology for aging in place: a systematic review. International journal of medical informatics. 2014;83(4):235–48. 10.1016/j.ijmedinf.2014.01.004 24529817 10.1016/j.ijmedinf.2014.01.004

[101] Anstey KJ, Eramudugolla R, Dixon RA. Contributions of a risk assessment approach to the prevention of alzheimer's disease and dementia. Journal of Alzheimer's Disease. 2014;42:S463–S73. 10.3233/JAD-141248 25114084 10.3233/JAD-141248

[102] Finlay L. “Outing” the researcher: The provenance, process, and practice of reflexivity. Qualitative health research. 2002;12(4):531–45. 10.1177/104973202129120052 11939252 10.1177/104973202129120052

[103] Tong A, Sainsbury P, Craig J. Consolidated criteria for reporting qualitative research (COREQ): a 32-item checklist for interviews and focus groups. International journal for quality in health care. 2007;19(6):349–57. 10.1093/intqhc/mzm042 17872937 10.1093/intqhc/mzm042

