# Supplementary material for: Experiences of mobile health in promoting physical activity: A qualitative systematic review and meta-ethnography
Source: PLoS One. 2018 Dec 17;13(12):e0208759. doi: 10.1371/journal.pone.0208759 (PMC6296673; doi:10.1371/journal.pone.0208759)
Supplement: S2 File — (DOCX) [file pone.0208759.s002.docx]

S2 File. Medline Search Terms.

| **Combined using AND** | **Key concepts** | **Search terms** |
| --- | --- | --- |
|  | Qualitative research | qualitative OR “patient experience” OR “patients’ experiences” OR “patient perception” OR “patients’ perceptions” OR “patient perspective” OR “patients’ perspectives” OR “case studies” OR “evaluation methods” OR interview* OR “focus group*” OR “mixed methods” OR “naturalistic observation” OR “participant observation” OR “social science research” OR “nursing methodology research” OR “consumer participation” OR transcript* OR ethnograph* OR phenomenol* OR ethnonurs* OR “grounded theor*” OR “grounded-theor*” OR “purposive sample” OR hermeneutic* OR heuristic* OR semiotics OR “lived experience*” OR narrative* OR “life experience*” OR “life stor*” OR “cluster sample” OR “action research” OR “observational method” OR “content analysis” OR “thematic analysis” OR “narrative analysis” OR “constant comparative method” OR “field stud*” OR “field-notes” OR “audiorecording” OR “videorecording” OR “theoretical sample” OR “discourse analysis” OR “ethnological research” OR ethnomethodolog* OR (MH "interviews as topic") OR (MH "focus groups") OR (MH "narration") OR (MH "qualitative research") |
|  | mHealth | mhealth OR m-health OR “m health” OR “mobile health” OR “mobilehealth” OR “mobile-health” OR “mobile phone” OR mobile OR “mobile electronic devic*” OR smartphone OR “handheld computer” OR “cellular phone” OR “cell phone” OR iphone OR “i phone” OR ipod OR “i pod” OR ipad OR “i pad” OR tablet OR SMS OR “text messag*” OR “short message” OR “multimedia messag*” OR “multi-media messag*” OR “multimedia messag*” OR “mobile technology” OR “mobile communication” OR “mobile app*” OR “MP3 player*” OR “wearable sensor” OR “body worn sensor” OR smartwatch OR accelerometer OR “mobile computing” OR “personal digital assistant*” OR “wireless devic*” OR “global positioning system” OR “bluetooth technolog*” OR “mobile operating system” OR apple OR android OR ios OR blackberry OR windows OR samsung OR nokia OR “social media” OR “social network*” OR (MH "Telemedicine+") |
|  | Physical activity | “physical activit*” OR exercis* OR fitness OR “energy expenditure” OR walk* OR run* OR cycl* OR bicycl* OR jog* OR swim* OR “aquatic exercis*” OR sport OR “aerobic exercis*” OR “resistance training” OR “muscle stretching exercis*” OR pilates OR “Tai chi” OR yoga OR “strength training” OR “weight lifting” OR “strengthening program” OR “weight bearing” OR “isometric exercise*” OR “motor control exercis*” OR “range of motion exercis*” OR “range-of-motion exercis*” OR “flexibility exercis*” OR “balance training” OR “proprioceptive training” OR “circuit-based exercis*" OR "cool-down exercis*" OR "warm-up exercis*" OR "physical conditioning” OR “plyometric exercis*” OR (MH "Physical Activity") |

**Limits applied: humans and adults.**
